# Overexpression of Nodal induces a metastatic phenotype in pancreatic cancer cells via the Smad2/3 pathway

**DOI:** 10.18632/oncotarget.2686

**Published:** 2015-02-12

**Authors:** Wanxing Duan, Rong Li, Jiguang Ma, Jianjun Lei, Qinhong Xu, Zhengdong Jiang, Ligang Nan, Xuqi Li, Zheng Wang, Xiongwei Huo, Liang Han, Zheng Wu, Erxi Wu, Qingyong Ma

**Affiliations:** ^1^ Department of Hepatobiliary Surgery, First Affiliated Hospital, Xi'an Jiaotong University, Xi'an 710061, China; ^2^ Department of Pediatrics, Tongji Hospital, Tongji Medical College, Huazhong University of Science and Technology, Wuhan 430030, China; ^3^ Department of Oncology, First Affiliated Hospital, Xi'an Jiaotong University, Xi'an 710061, China; ^4^ Department of General Surgery, First Affiliated Hospital, Xi'an Jiaotong University, Xi'an 710061, China; ^5^ Department of Pharmaceutical Sciences, North Dakota State University, Fargo, North Dakota 58105, USA

**Keywords:** Nodal, metastasis, pancreatic cancer, Smad2/3 pathway

## Abstract

Metastasis is the major cause for the high mortality rate of pancreatic cancer. Human embryonic stem cell (hESC) associated genes frequently correlate with malignant disease progression. Recent studies have demonstrated that the embryonic protein Nodal, which plays a critical role during embryonic development, is re-expressed in several types of tumors and promotes cancers progression. However, little is known about the role of Nodal in pancreatic cancer. Here, we show that Nodal expression is upregulated in human pancreatic cancer tissues. Moreover, Nodal expression levels correlate well with the grade of pancreatic cancer differentiation. In addition, we present clear evidence that Nodal induces signal transduction through the Smad2/3-dependent pathway *in vitro*. Furthermore, we show that Nodal promotes pancreatic cancer cell migration and invasion, induces epithelial-mesenchymal transition (EMT) and enhances the expression of matrix metalloproteinase-2 (MMP2) and CXC chemokine receptor 4 (CXCR4). Using an *in vivo* liver metastasis model of pancreatic cancer, we observed that blocking Nodal signaling activity with the small-molecule inhibitor SB431542 decreases the number and size of liver metastases. Taken together, our results suggest that Nodal overexpression induces a metastatic phenotype in pancreatic cancer cells, and that targeting Nodal signaling may be a promising therapeutic strategy for pancreatic cancer.

## INTRODUCTION

Pancreatic cancer is one of the most lethal cancers, causing an estimated 227000 deaths per year worldwide [1]. In particular, pancreatic ductal adenocarcinoma (PDAC) is the most common histological type with a highly invasive and metastatic phenotype that is often responsible for treatment failure and an extremely poor clinical prognosis. To improve patient survival, understanding the regulatory molecular mechanisms that control the metastasis of PDAC is important.

Invasive cancer cells share some similar properties with human embryonic stem cells (hESCs) [2]. For example, cancer cell proliferation, self-renewal, and epithelial-mesenchymal transition (EMT) are defined features of hESCs. EMT generally occurs during embryonic development and is also an important element in cancer progression, endowing cells with migratory and aggressive properties that consequently lead to tumor metastasis [3]. Accordingly, several studies have demonstrated that hESC-associated genes highly correlate with malignant disease initiation and progression [4, 5]. Nodal is a potent embryonic morphogen from the transforming growth factor (TGF)-β family that plays critical roles during embryonic development, including regulating dorsal mesoderm induction, anterior patterning and left–right asymmetry formation [6–8]. Nodal also has an important role in maintaining the self-renewal capacity and pluripotency of hESCs [9, 10]. In embryos, Nodal protein functions by binding to activin-like kinase receptors type I (ALK4/7) and type II (ActRIIB) and the co-receptor Cripto-1, triggering downstream phosphorylation of Smad2 and Smad3 (Smad2/3) to regulate target gene expression [11]. Generally, Nodal expression is relatively restricted to embryonic tissues and hESCs and is barely detectable in most normal adult tissues. However, recent studies have shown that Nodal is aberrantly upregulated in melanoma, glioma, breast cancer, prostate cancer and endometrial cancer [2, 12–15]. Importantly, Nodal expression in malignancies correlates with cancer growth and progression and may be a prognostic marker [16]. These studies demonstrated that abnormally expressed Nodal promotes cancer cell proliferation, invasion, migration and inhibits apoptosis; moreover, Nodal induces angiogenesis by accelerating VEGF and PDGF expression and secretion [17–19]. In a normal adult pancreas, the Nodal gene is not expressed except for during pancreatic islet regeneration [20]. Similarly, the reactivation of Nodal signaling might have important functional consequences for pancreatic cancer development and progression. However, little is known about Nodal in pancreatic cancer. Although Heeschen and colleagues have revealed that Nodal/Activin signaling drives the self-renewal and tumorigenicity of pancreatic cancer stem cells (CSCs), CSCs are a rare subpopulation, accounting for only 0.2–0.8% of pancreatic cancer cells [21, 22]. Whether Nodal is expressed widely in pancreatic cancer cells or impacts the behavior of the majority of pancreatic cancer cells is ill-defined.

EMT is a process during which cells lose their polarized epithelial traits and acquire mesenchymal characteristics such as the downregulation of E-cadherin and the upregulation of N-cadherin and Vimentin, consequently inducing an aggressive phenotype [3, 23]. EMT plays a pivotal role in cancer metastasis. This crucial event has been associated with the overexpression of several EMT-inducing transcription factors, such as Snail, a zinc finger transcription repressor [24]. Increased expression of matrix metalloproteinases (MMPs) with the capacity for extracellular matrix (ECM) degradation is known to play an important role in cancer angiogenesis, invasiveness and metastatic potential [25, 26]. One of the MMPs of particular significance in tumor contexts is MMP2. Furthermore, CXC chemokine receptor 4 (CXCR4), which selectively binds the CXC chemokine stromal cell-derived factor 1 (SDF-1), correlates well with malignant progression, especially distant metastasis in a variety of human tumors including PDAC [27, 28]. CXCR4-positive tumor cells might migrate toward distant organs in response to an SDF-1 gradient. Moreover, we have demonstrated that SDF-1/CXCR4 signaling induces pancreatic cancer cell invasion and EMT through non-canonical activation of the Hedgehog pathway [29].

In this study, we focused on exploring Nodal expression and its role in pancreatic cancer progression. We found that Nodal expression is upregulated widely in pancreatic cancer cells, not only in CSCs, but also in tumor-associated stromal cells, and it induces a metastatic phenotype in pancreatic cancer cells by promoting EMT and enhancing the expression of MMP2 and CXCR4 via the Smad2/3 pathway, indicating that Nodal signaling is a critical mechanism in the metastasis of pancreatic cancer and might be a therapeutic target for the treatment of pancreatic cancer.

## RESULTS

### Expression of Nodal in pancreatic cancer tissues

**Figure 1:**
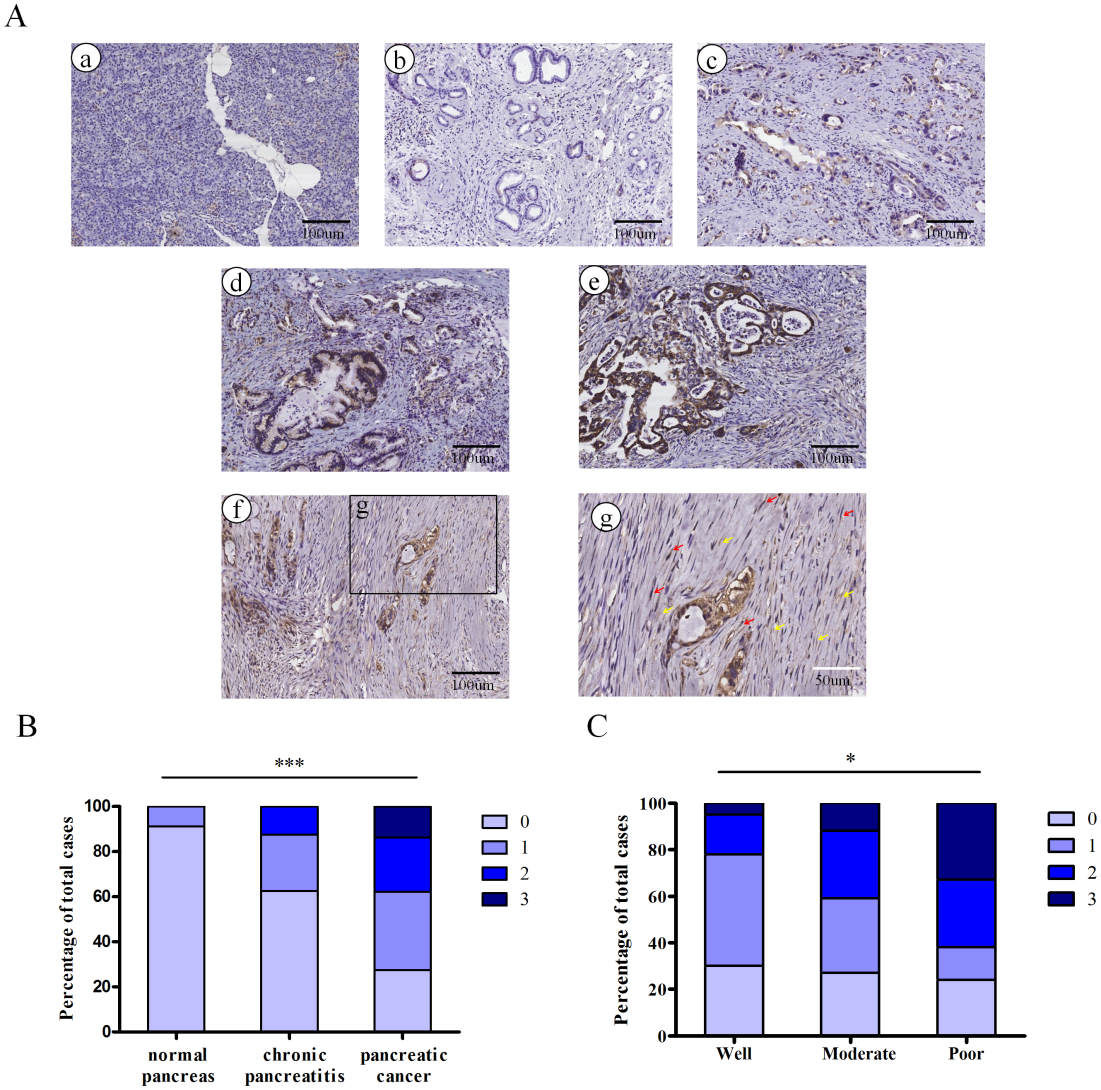
The embryonic protein Nodal is expressed in pancreatic ductal adenocarcinoma **(A)** Representative images of normal pancreas (a) and of pancreatic cancers with absent (b), weak (c), moderate (d) and strong (e) Nodal staining (brown) are shown. Representative images of Nodal expression in tumor-associated stromal tissues (f and g) including stromal cells (red arrows) and ECM (yellow arrows) in specimens with moderate and strong staining. Black scale bars, 100 μm; white scale bars, 50 μm. **(B)** Nodal expression was markedly increased in PDAC compared with non-tumor tissues (*P* < 0.0001). **(C)** Nodal expression in poorly differentiated pancreatic cancer tissues is significantly stronger compared to well-differentiated pancreatic cancer tissues (*P* = 0.0277). Data are from the analysis of 142 pancreatic tissue specimens.

To evaluate Nodal expression, pancreatic tissue sections from 142 patients identified as normal pancreas, chronic pancreatitis or PDAC were analyzed using immunohistochemistry. The statistics for Nodal expression levels in different pancreatic tissue groups are shown in Supplementary Table S1. Figure 1 shows representative pictures of absent (0; Figure 1Ab), weak (1+; Figure 1Ac), moderate (2+; Figure 1Ad) and strong (3+; Figure 1Ae) Nodal staining in pancreatic cancer. As shown in Figure 1Aa, little or no Nodal immunoreactivity was observed in normal pancreatic tissues. Nodal expression was significantly increased in PDAC compared to non-tumor tissues (*P* < 0.0001; Figure 1B). Moreover, tumor-associated stromal tissue including stromal cells and ECM also expressed Nodal when the pancreatic cancer cells exhibited moderate to strong staining (Figure 1Af and 1Ag, Supplementary Table 2). Notably, Nodal expression levels correlated well with the grade of pancreatic cancer differentiation, with stronger Nodal expression in poorly differentiated pancreatic cancer tissues compared to well-differentiated pancreatic cancer tissues (*P* = 0.0277; Figure 1C).

### Expression of Nodal in pancreatic cancer cell lines and pancreatic stellate cells

**Figure 2:**
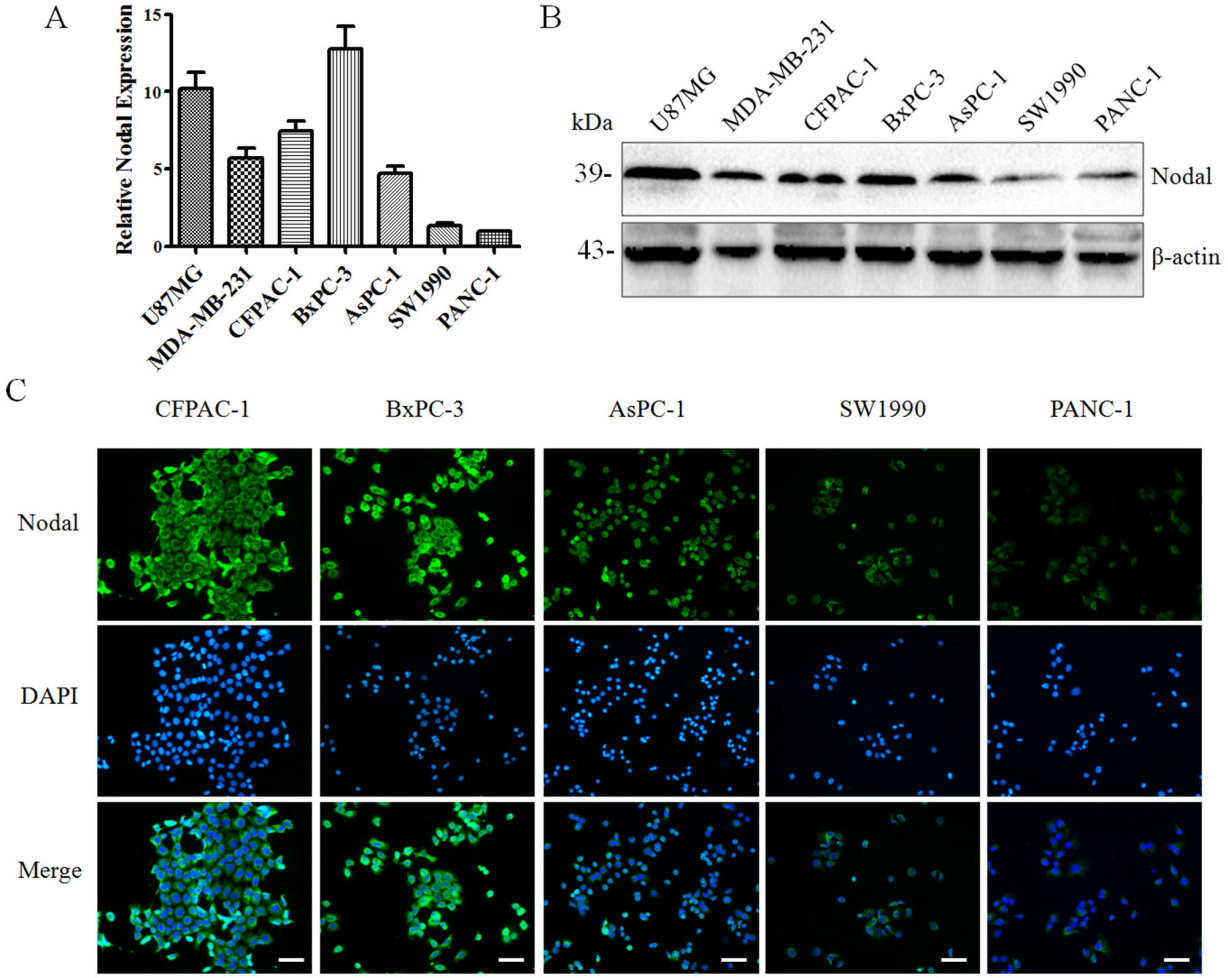
The expression of Nodal in human pancreatic cancer cell lines Using real-time PCR **(A)** and Western blotting **(B)**, the mRNA and protein expression levels of Nodal were detected in a panel of human pancreatic cancer cell lines using U87 MG and MDA-MB-231 cells as the positive controls. **(C)** Immunofluorescence confirming the findings presented in (A&B) and also indicating that Nodal was expressed in the cytoplasm of the majority of pancreatic cancer cells rather than only in CSCs. White scale bars, 50 μm. All of the data are presented as the mean ± SD of three independent experiments performed in triplicate.

To identify appropriate models to explore the possible roles of Nodal in pancreatic cancer, the Nodal expression level in five human pancreatic cancer cell lines was evaluated. U87 MG and MDA-MB-231 cells, which have both identified as cell lines with high Nodal expression, were used as positive controls [12, 14]. At the mRNA and protein levels, we showed that all of these cell lines express Nodal and observed higher levels in CFPAC-1and BxPC-3 cells. In addition, SW1990 and PANC-1 cells exhibited lower expression levels (Figure 2A and 2B). Using immunofluorescence, we further verified these results and revealed that Nodal is expressed in the cytoplasm (Figure 2C). Moreover, we observed that Nodal protein is expressed widely in pancreatic cancer cells rather than restricted to a subpopulation of CSCs (Figure 2C). Accordingly, we defined BxPC-3 as a high-expression sample and PANC-1 as a low-expression sample for further experiments.

**Figure 3:**
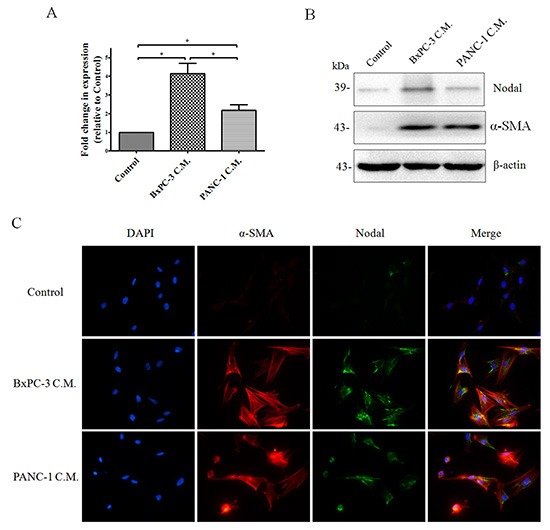
The expression of Nodal in human pancreatic stellate cells (PSCs) Subconfluent PSCs isolated from normal pancreas tissues were treated with BxPC-3 or PANC-1 cell conditioned media as described in the Materials and Methods for 3days. Real-time PCR **(A)** and Western blotting **(B)** to detect Nodal expression in PSCs were performed. PSCs cultured in cancer cell C.M. displayed markedly increased Nodal expression compared to the control media. (B) Western blotting of a-SMA protein further confirming the identity of PSCs. **(C)** Immunofluorescence of Nodal and a-SMA protein in PSCs confirming the finding presented in (A&C). Magnification, × 400. C.M., conditioned media. *P < 0.05. All of the data are presented as the mean ± SD of three independent experiments performed in triplicate.

Previous research indicated that activated pancreatic stellate cells (PSCs) express Nodal and form an ideal microenvironment for pancreatic cancer stem cells via Nodal signaling [30]. Unfortunately, the PSCs investigated in that study were isolated and immortalized from chronic pancreatitis patient and were more appropriate for the examination of pancreatic fibrosis. As shown in Figure 3A, 3B and 3C, we found that quiescent PSCs isolated from a normal pancreas express little Nodal. However, after incubating the cells with cancer cell conditioned media containing Nodal protein (Supplementary Figure 2) for 3 days, PSCs showed a marked increase in Nodal expression compared to the untreated control. Furthermore, the effect of conditioned media from BxPC-3 cells was stronger compared to that from PANC-1 cells. In addition, as shown in Supplementary Figure 3, the cancer cell conditioned media induced Nodal upregulation in PSCs could be efficiently abrogated by Actinomycin D (a transcription inhibitor), indicating that the Nodal protein in treated PSCs was not internalized. Taken together, Nodal proteins were expressed by both pancreatic cancer cells and tumor-associated PSCs.

### Nodal signaling in pancreatic cancer cells activates Smad2/3 pathway

Nodal is a novel member of the TGF-β superfamily that binds to activin-like kinase type II and type I receptors and induces signal transduction through the Smad2/3 or Smad1/5/8 pathway [31]. In hESCs and other types of cancer cells, studies have demonstrated that Nodal drives signal transduction mainly via the Smad2/3 pathway [11]. In addition, Cripto-1, a member of the EGF-CFC family, acts as a co-receptor for Nodal [32].

**Figure 4:**
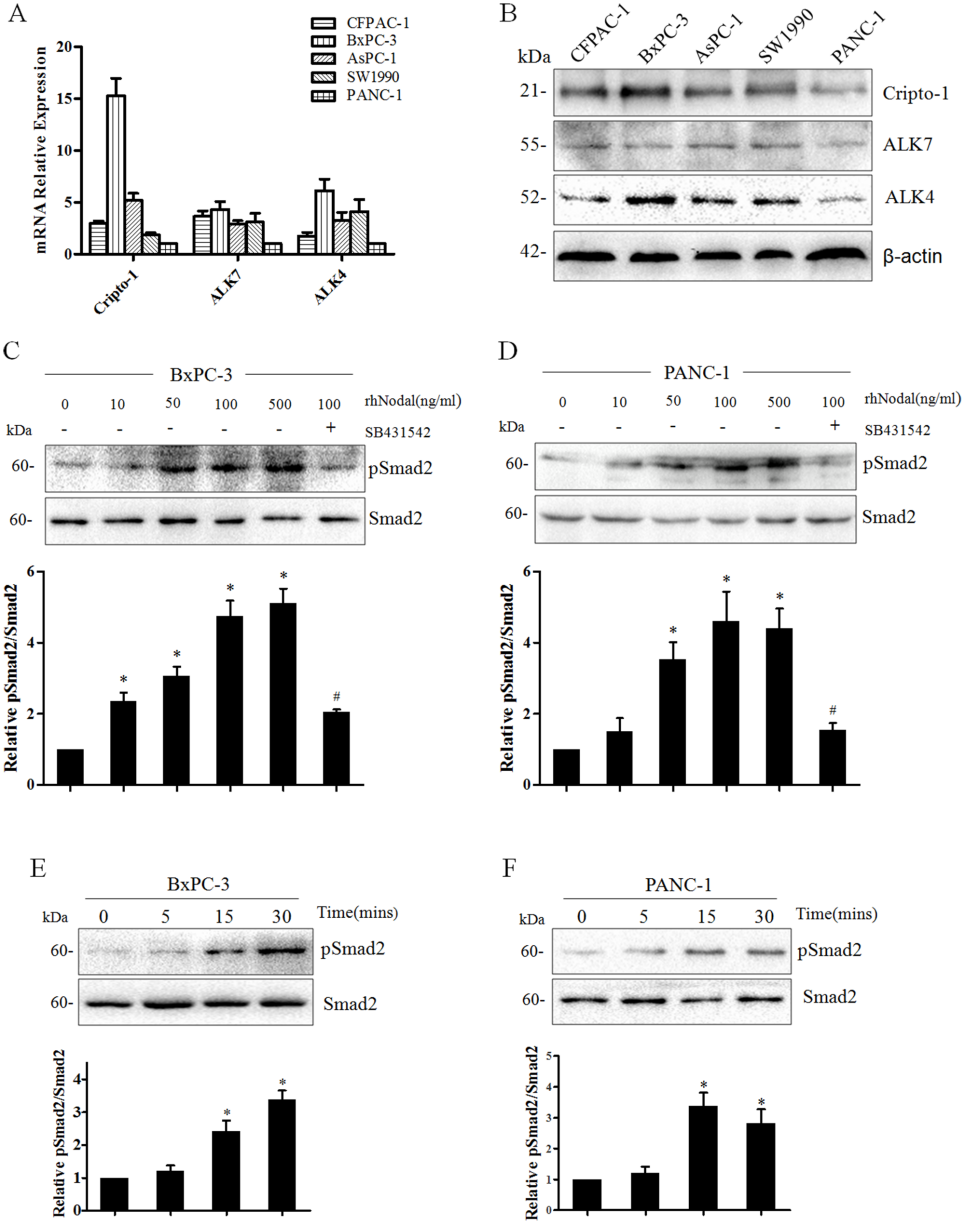
Nodal signaling in human pancreatic cancer cell lines **(A)** Real-time PCR analysis of Nodal signaling type I activin-like kinase receptors (ALK4/7) and its co-receptor Cripto-1 in a panel of human pancreatic cancer cell lines. β-actin was used to normalize the RNA inputs. **(B)** Western blotting analysis of ALK4/7 and Cripto-1 in human pancreatic cancer cell lines. β-actin served as a loading control. **(C&D)** Nodal protein induces the phosphorylation of Smad2 in both BxPC-3 and PANC-1 cells in a dose-dependent manner. BxPC-3 and PANC-1 cells were treated for 30 min with 10 - 500 ng/ml rhNodal and 10 μM SB431542. pSmad2 and total Smad2 levels were quantified from Western blotting using densitometry determined with QuantityOne image analysis software. **(E&F)** Western blotting analysis of pSmad2 and total Smad2 levels at different times in BxPC-3 and PANC-1 cells after stimulation with rhNodal (100 ng/ml). *P < 0.05 compared to the untreated group. #*P* < 0.05 compared to the treatment with 100 ng/ml rhNodal. All of the data are presented as the mean ± SD of three independent experiments performed in triplicate.

To confirm that the signaling in pancreatic cancer cells was responsive to upregulation of Nodal, we first detected the expression of the type I (ALK4/7) receptor and its co-receptor Cripto-1. As shown in Figure 4A and 4B, the mRNA and protein expression of these receptors were detected in all of human pancreatic cancer cell lines, including BxPC-3 and PANC-1 cells. This suggested that these cell lines have the potential to respond to Nodal, and BxPC-3 and PANC-1 cells were treated with different concentration of rhNodal. We observed that the phosphorylation of Smad2 in both BxPC-3 and PANC-1 cells occurred in a dose-dependent manner in response to Nodal (Figure 4C and 4D). Interestingly, 100 ng/ml appears to be the relevant concentration range, because higher levels of signaling were not observed even after treatment with more rhNodal. Moreover, SB431542, a specific molecular inhibitor of Nodal signaling, blocked the Nodal-induced Smad2 phosphorylation (Figure 4C and 4D). Furthermore, stimulation with 100 ng/ml rhNodal for different time periods also increased the basal levels of Smad2 phosphorylation in both BxPC-3 and PANC-1 cells (Figure 4E and 4F). These results indicated that pancreatic cancer cells respond to Nodal protein via the Smad2/3 pathway.

### Nodal enhances pancreatic cancer cells migration and invasion

**Figure 5:**
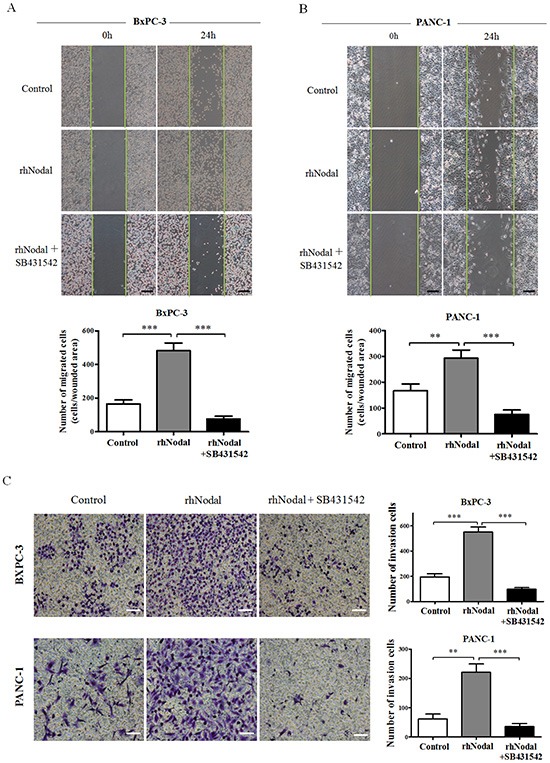
Nodal enhances pancreatic cancer cell migration and invasion **(A&B)** Wound healing assays were performed in BxPC-3 and PANC-1 cells pretreated with 100 ng/ml rhNodal or nothing (control) with or without the inhibitor SB431542 (10 μM) for 2 days. Images were visualized at time 0 h and 24 h. Quantitative analysis was carried out as described in the Materials and Methods. **(C)** Transwell chamber assays of BxPC-3 and PANC-1 cells. The pretreated cells were seeded into a Matrigel-coated invasion chamber for an additional 36 h. The invasive cells were quantified by counting the number of cells in 10 random fields. **P < 0.01; ***P < 0.001. Columns, mean; bars, SD. Black scale bars, 100 μm. White scale bars, 50 μm. All of the data are representative of at least three independent experiments.

To study the functional relevance of Nodal in pancreatic cancer cells, wound-induced migration assays quantified by counting cells migrating into the wounded area 24 h after scratching were performed under serum-free conditions. Figure 5 depicts representative images visualized at 0 h and 24 h. As shown in Figure 5A and 5B, both BxPC-3 and PANC-1 cells treated with 100 ng/ml rhNodal migrated more than control cells (*P* = 0.0004, *P* = 0.0057, respectively). Moreover, the significant increase in migration was limited by the inhibitor SB431542 in BxPC-3 and PANC-1 cells (*P* = 0.0001, *P* = 0.0004, respectively). Because the assays were performed in the absence of growth factors or serum, closing of the wounded area occurred by cell migration and not as a result of proliferation of cells at the wound edge. Additionally, Transwell invasion assays were also performed under serum-free conditions. rhNodal-treated BxPC-3 and PANC-1 cells showed significantly stronger invasion properties compared to control cells (*P* = 0.0002, *P* = 0.0010, respectively), which was also prevented by SB431542 (*P* < 0.0001, *P* = 0.0004, respectively) (Figure 5C). These data suggest that Nodal markedly stimulates pancreatic cancer cell motility.

### Nodal signaling induces EMT and enhances the expression of MMP2 and CXCR4

**Figure 6:**
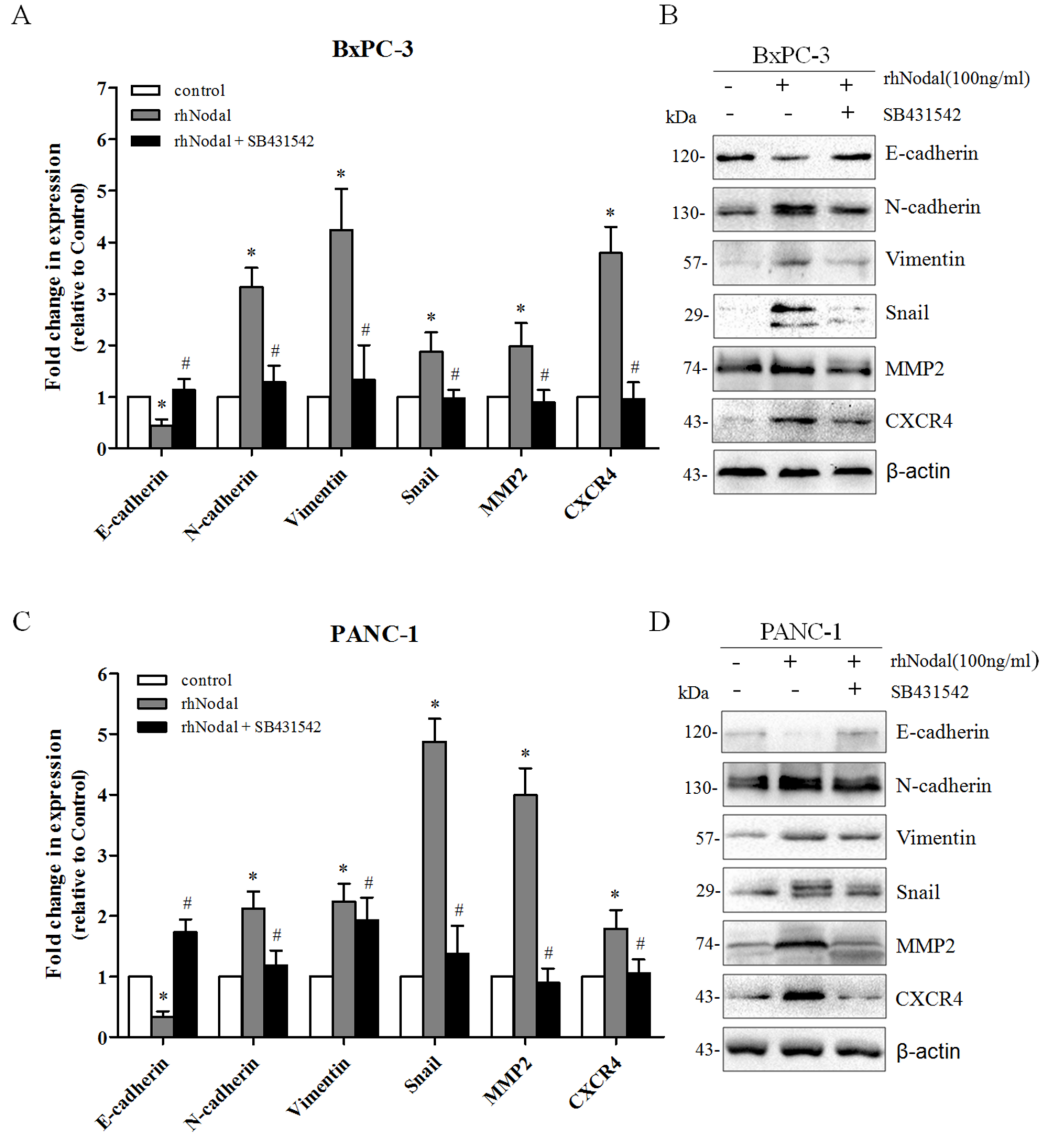
Nodal signaling induces EMT and enhances the expression of MMP2 and CXCR4 **(A&C)** Real-time PCR analysis of EMT markers, MMP2 and CXCR4 in BxPC-3 and PANC-1 cells exposed to 100 ng/ml rhNodal with or without 10 μM SB431542. The celltreated with 100 ng/ml rhNodal displayed a significant downregulation of the epithelial marker E-cadherin but an upregulation of the mesenchymal markers N-cadherin, Vimentin and the transcription factor Snail, and an increase in MMP-2 and CXCR4 expression compared to untreated cells. Treatment with SB431542 (10 μM) blocked thegene expression changes induced by rhNodal (100 ng/ml). **(B&D)** Western blotting analysis verified the changes described in (A&C). β-actin was used as an internal loading control. All of the data are presented as the mean ± SD of three independent experiments performed in triplicate. *P < 0.05 compared to the untreated group. #*P* < 0.05 compared to treatment with 100 ng/ml rhNodal.

In cancer contexts, cellular migration and invasive properties are frequently correlated with EMT and MMPs. To explore the underlying mechanisms of Nodal-elevated cancer cell motility, we detected the expression of key markers of the EMT process and MMP-2 in BxPC-3 and PANC-1 cells after treatment with rhNodal and SB431542. As shown in Figure 6, at the mRNA and protein levels, we validated that addition of rhNodal (100 ng/ml) resulted in a significant downregulation of the epithelial marker E-cadherin and an upregulation of the mesenchymal markers N-cadherin and Vimentin, the transcription factor Snail, and a significant increase in MMP-2 expression in both BxPC-3 (Figure 6A and 6B) and PANC-1 cells (Figure 6C and 6D). In addition, we observed that treatment of BxPC-3 and PANC-1 cells with 100 ng/ml rhNodal dramatically upregulated CXCR4 expression (Figure 6). Furthermore, blocking Nodal signaling with SB431542 treatment (10 μM) prevented the changes induced by 100 ng/ml rhNodal (Figure 6).

### Loss of Nodal expression reverses the invasive phenotype of pancreatic cancer cells

**Figure 7:**
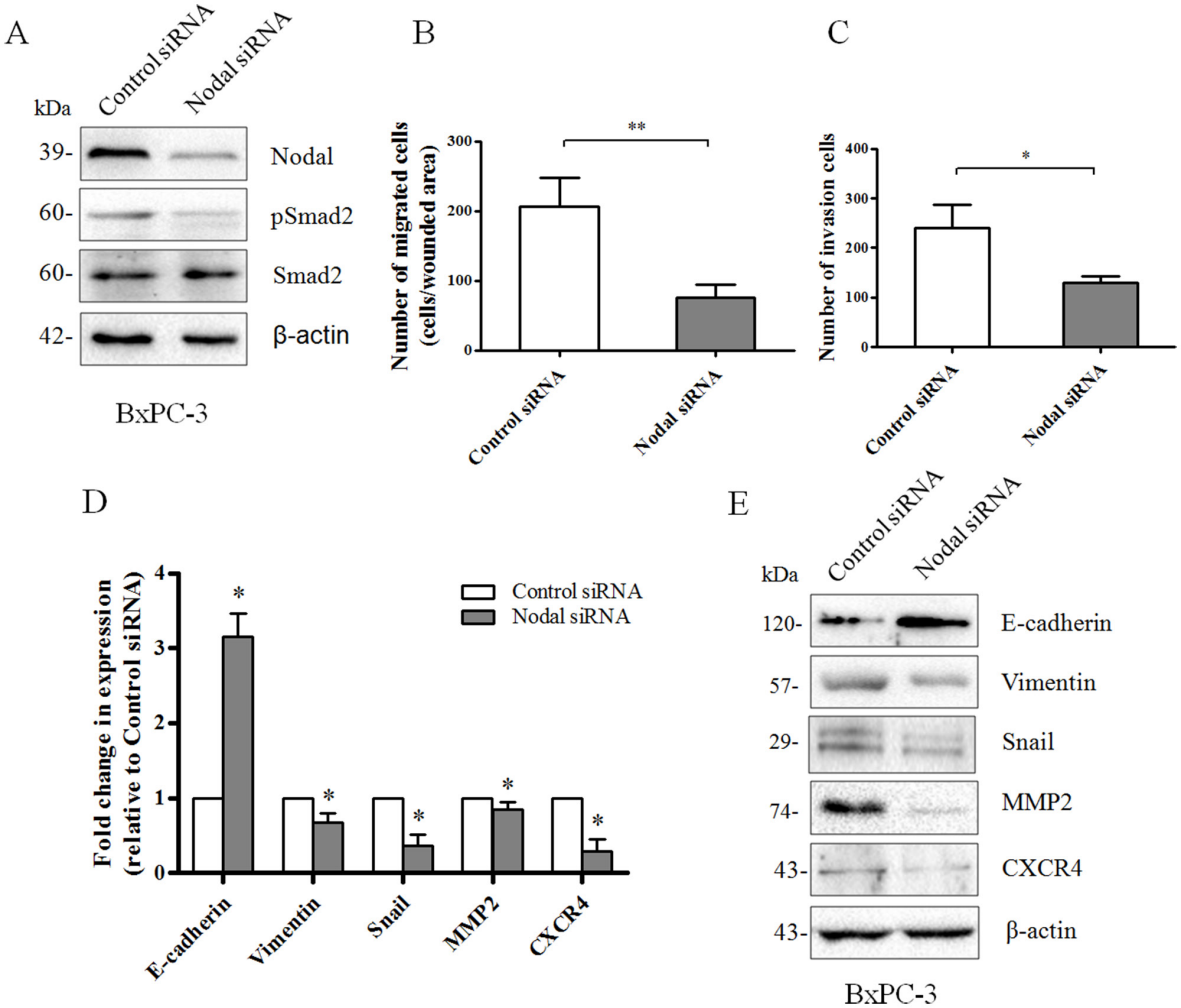
Loss of Nodal expression reverses the invasion phenotype of pancreatic cancer cells **(A)** Interference with Nodal expression in BxPC-3 cells was analyzed by Western blotting. Knockdown of Nodal reduced the phosphorylation of Smad2. β-actin was used as an internal loading control. **(B)** Wound-healing assays indicating that knocking down Nodal expression significantly reduced the migration of BxPC-3 cells (*n* = 3, *P* = 0.0074). **(C)** Transwell chamber assays with Matrigel indicating that knocking down Nodal expression significantly reduced invasion of BxPC-3 cells (*n* = 3, *P* = 0.0156). **(D)** Real-time PCR analysis revealing that BxPC-3 cells transfected with a Nodal-targeted siRNA markedly decreased the expression of N-cadherin, Vimentin, Snail, MMP-2 and CXCR4and significantly increased the expression of E-cadherin compared to a scramble control siRNA. **(E)** Western blotting analysis confirming the changes described in (D). β-actin was used as an internal loading control. All of the data are presented as the mean ± SD of three independent experiments performed in triplicate. *P < 0.05; ** *P* < 0.01 compared to control siRNA.

To confirm the above results, we knocked down Nodal expression with siRNA technology in BxPC-3 cells which is the Nodal high-expression sample (Figure 7A). We found that knockdown of Nodal reduced the phosphorylation of Smad2 (Figure 7A). Using wound-healing assays, we found that knocking down Nodal expression significantly reduced the migration of BxPC-3 cells (Figure 7B). Using a Transwell chamber assays with Matrigel, a significant decrease in the migration of siNodal BxPC-3 cells was observed compared to siControl cells (Figure 7C). Furthermore, real-time PCR verified that silencing of Nodal resulted in a marked decrease in the expression of N-cadherin, Vimentin and Snail, but a significant increase of E-cadherin (Figure 7D), consistent with reversion to an epithelial phenotype. These observations were confirmed at the protein level by Western blotting (Figure 7E). At the mRNA and protein levels, we demonstrated that knockdown of Nodal significantly decreased the expression of MMP2 and CXCR4. Together, these data suggested that Nodal signaling might participate in the metastatic process of PDAC.

### Inhibition of Nodal signaling *in vivo* by SB431542 administration reduces distant metastasis of pancreatic cancer

**Figure 8:**
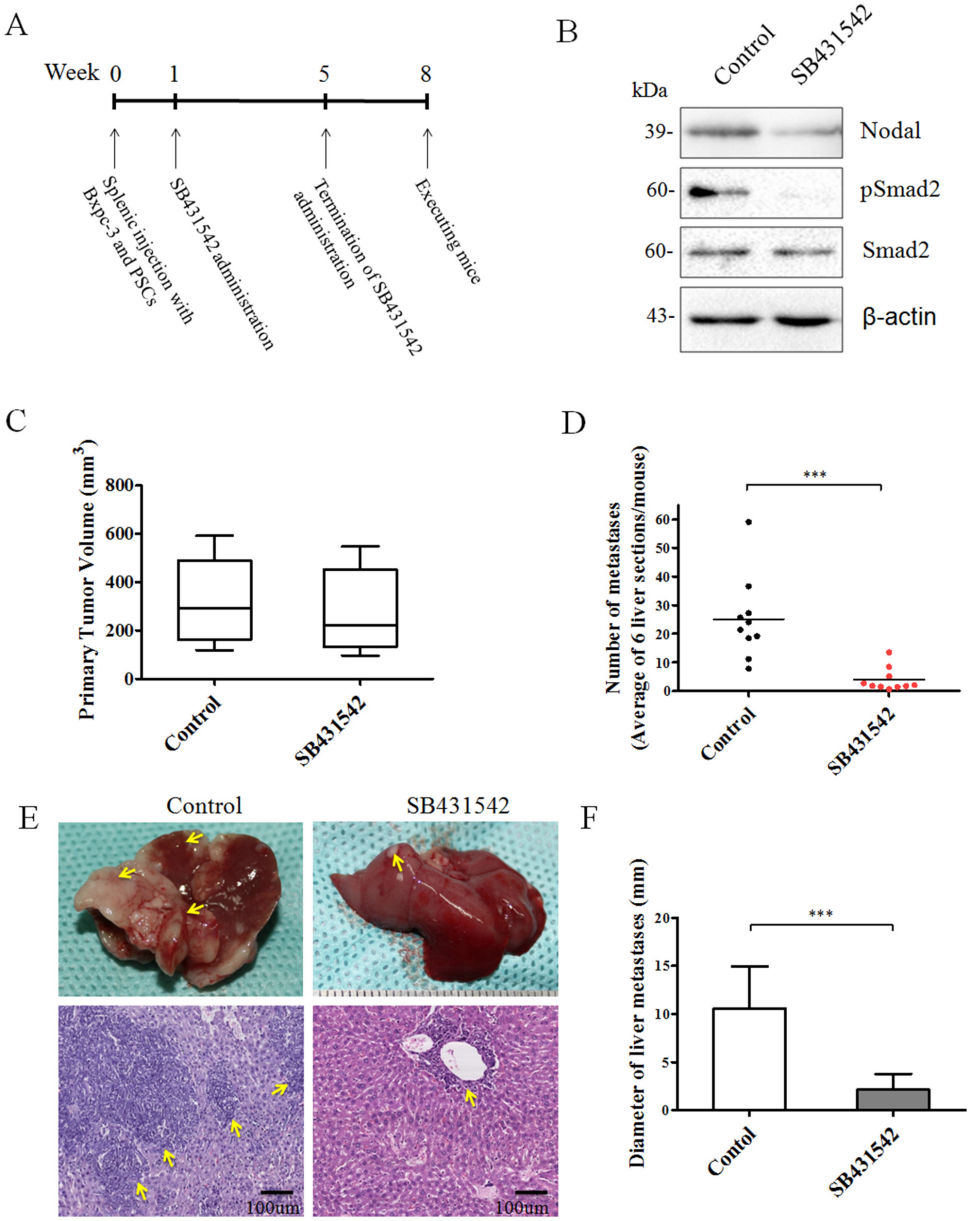
Effect of Nodal signaling inhibition by SB431542 administration *in vivo* **(A)** Experimental procedures for *in vivo* experiments were described in the Materials and Methods. **(B)** Western blotting analysis of primary tumor tissues from two groups demonstrating that SB431542 administration efficiently downregulated Nodal expression and blocked Nodal signaling. **(C)** Box plot showing the primary tumor size of mice from each group. SB431542 treatment did not statistically significantly decelerate primary tumor growth compared to the untreated control (*P* = 0.5631). The numbers **(D)** and sizes **(F)** of metastatic tumors in the liver were measured as described in the Materials and Methods. **(E)** Representative image of liver metastases in the two groups. Lower panel shows a HE section from the control and SB431542 groups. Arrows indicate the location of liver metastases (*n* = 10). ***P < 0.001. Data are represented as mean ± SD.

Based on the above promising findings, we determined whether inhibition of Nodal signaling reduces the distant metastasis of pancreatic cancer. To establish a reliable liver metastasis model, splenic injection with a BxPC-3 and PSCs mixed single-cell suspension (cell proportion 5:1) was performed, and treatment with SB431542 was initiated 1 week after inoculation as described in the Materials and Methods (Figure 8A). In previous reports, SB431542 administration *in vivo* efficiently inhibited the Nodal signaling pathway and downregulated Nodal expression [21]. In this experiment, Nodal expression in the SB431542-treated group was obviously decreased compared to the control group (Figure 8B). The phosphorylation of Smad2 in the treated group was decreased compared to the untreated control (Figure 8B), suggesting that SB431542 administration efficiently blocked Nodal signaling. The average size of primary tumors in the two groups was assessed 8 weeks after inoculation. SB431542 treatment did not statistically significantly decelerate primary tumor growth (*P* = 0.5631compared to the untreated group) (Figure 8C). As shown in Supplementary Figure 4, metastases tumor tissues exhibited higher Nodal expression compared to primary tissues, and SB431542 administration efficiently down regulated Nodal expression in metastases tumor specimens, indicating that Nodal signaling might be associated with the process of metastasis. Then, we examined the number and size of the liver metastases. Compared to the control group treated with PBS, administration of SB431542 significantly decreased the number (*n* = 10, *P* = 0.0003; Figure 8D) and size (*n* = 10, *P* < 0.0001; Figure 8F) of the liver metastases.

## DISCUSSION

Current therapies for managing pancreatic cancer patients are largely ineffective. The major reason for the extremely high mortality rate in pancreatic cancer patients is the invasive and metastatic phenotype of the cancer cells. Previous studies have demonstrated that hESC-associated genes are re-expressed in tumor tissue and developmental signaling pathways are re-activated during malignant disease initiation and progression [2, 4, 5]. In this study, we explored the role of Nodal, which is a potent embryonic morphogen, in the invasiveness and metastasis of pancreatic cancer cells for the first time.

Our study showed that Nodal expression was highly upregulated in pancreatic cancer tissues compared to non-tumor tissues. Moreover, Nodal expression level correlated well with the grade of pancreatic cancer differentiation. A previous study revealed that Nodal is overexpressed in pancreatic cancer stem cells (CSCs) and drives the self-renewal and tumorigenicity of CSCs [21]. Here, we observed that Nodal expression was widely detected in both pancreatic cancer tissue specimens and cell lines rather than only in CSCs. Using immunohistochemistry, we also found that Nodal expression was detected in the tumor-associated stromal tissue, including stromal cells and the ECM, in specimens with moderate to strong immunoreactivity. This finding is in accordance with detectable Nodal expression in breast cancer-associated stroma [16]. *In vitro*, we further demonstrated that tumor-associated PSCs, which are the critical stromal cells in pancreatic cancer, exhibit increased Nodal expression compared to quiescent PSCs. PSCs have been defined as the principal source of excessive extracellular matrix production in pancreatic cancer [33]. Given that we observed immunoreactivity in pancreatic cancer ECM, tumor-associated PSCs might secrete Nodal protein into the ECM. This agrees with a previous report demonstrating that PSCs immortalized from chronic pancreatitis patient promote pancreatic cancer stem cell self-renewal and invasiveness via Nodal/Activin signaling [30]. Taken together, these results support the notion that Nodal accumulates in pancreatic cancer tissues in both autocrine and paracrine manner.

In hESCs and other types of cancer cells, Nodal regulates target gene transcription by phosphorylating heterodimeric complexes of type I (ALK4/7) and type II (ActRIIB) activin-like kinase receptors followed by the ALK 4/7–mediated phosphorylation of Smad2/3, with or without the Smad4 phosphorylation [34], and translocates into the nucleus [2, 9, 12]. In addition, Cripto-1 is a prominent mediator of the Nodal signaling pathway [32]. In this study, we present clear evidence that Nodal induces signal transduction through the Smad2/3-dependent pathway. We confirmed that Alk4/7 and Cripto-1 were expressed in a panel of human pancreatic cancer cell lines, suggesting that these cell lines have the potential to respond to changes in Nodal expression. We showed that treatment with different concentrations of rhNodal increased Smad2 phosphorylation in BxPC-3 and PANC-1 cells. In contrast, pharmacologic inhibition of Nodal signaling by SB431542, as well as specific siRNA reduction of Nodal, efficiently suppressed Smad2 phosphorylation to pSmad2. Recent studies have shown that Nodal induces not only the Smad2/3 signaling pathway but also ERK1/2 signaling and PI3K/AKT signaling in breast cancer and melanoma, respectively [35, 36]. Further studies should focus on whether Nodal elevation in pancreatic cancer induces a Smad2/3-independent pathway.

The process of cancer metastasis is widely recognized as follows. When cells in the primary tumor sitealter their gene expression (called “reengineering”), the cancer cells become invasive and can penetrate the tumor stroma, entering the blood circulation or the lymphatic system via intravasation and forming second lesions. A comfortable pre-metastatic niche must be established for the travelling “seeds” forming macrometastases [37, 38]. The EMT process correlates well with cancer metastasis, inducing an aggressive phenotype when cancer cells lose their polarized epithelial traits and acquire mesenchymal characteristics [3, 23]. Secretion of MMP2, which shows strong proteolytic activity toward the ECM, enables cancer cells to gain increased motility, invade adjacent tissues and cross the endothelial barriers, consequently leading to tumor metastasis [25, 26]. The SDF-1/CXCR4 signaling axis participates actively in cancer metastasis [27, 28]. CXCR4-positive tumor cells might migrate toward distant organs in response to an SDF-1 gradient. In our study, we found that activation of Nodal signaling by the addition of rhNodal markedly enhanced pancreatic cancer cell migration and invasion. Conversely, inhibition of Nodal signaling by SB431542 or by knockdown of Nodal reduced Nodal-stimulated cell migration and invasion. Mechanistic investigation revealed that Nodal induced an aggressive phenotype in pancreatic cancer cells by initiating an EMT process and increasing MMP2 secretion. These results are consistent with previous studies observing that Nodal induces EMT and stimulates MMP2 secretion in breast cancer and melanoma [35, 36]. Through integrative genomic analyses, a previous study demonstrated that TGFβ/Nodal/Activin signaling based on Smad2/3 induces CXCR4 upregulation [39]. Here, we expanded this preliminary finding and verified that Nodal enhances CXCR4 expression in pancreatic cancer cells via the Smad2/3 pathway *in vitro*. We further demonstrated that blockade of Nodal signaling activity by SB431542 administration significantly reduces the number and size of liver metastases from human pancreatic cancer cells in a reliable experimental metastasis model but has very little effect on primary tumor growth. Given that Nodal signaling drives self-renewal and tumorigenicity of CSCs, which constitute a pivotal subpopulation of metastatic tumor cells [40, 41], the decrease in pancreatic cancer distant metastasis caused by inhibition of Nodal signaling might be explained by the suppression of cancer cells reengineering in the primary tumor site and by a reduction in CSCs viability. However, whether Nodal signaling is involved in the process of establishing a pre-metastatic niche in the second lesions should be further investigated. In accordance with previous reports that an autocrine loop of Nodal signaling might exist during the malignant progression of gliomas and hESCs [12, 42], we also observed that Nodal expression in primary tumor specimens of SB431542-treated nude mice was obviously lower than specimens from the control group.

## MATERIALS AND METHODS

These studies were approved by the relevant Ethical Committee of the First Affiliated Hospital of Medical College, Xi'an Jiaotong University, China.

### Collection of tissues and immunohistochemistry

One hundred forty-two paraffin-embedded pancreatic tissue samples including 23 normal samples, 24 chronic pancreatitis samples and 95 PDAC samples were obtained from the First Affiliated Hospital of Medical College, Xi'an Jiaotong University. PDAC patients with a history of chemotherapy or radiation therapy before sampling were excluded from this study. All samples were pathologically confirmed. In addition, the histological differentiation of PDAC samples was graded by experienced pancreatic pathologists. Immunohistochemical staining of Nodal was performed as previously described [13, 16] by incubationwith a mouse anti-Nodal antibody (Abnova, Taipei, Taiwan). A chromogenic reaction using 3,3-diaminobenzidine and a counterstain with hematoxylin were used to visualize staining. As a negative control, adjacent serial specimens were incubated with a normal mouse IgG control (Jackson ImmunoResearch Laboratories, West Grove, PA). Nodal staining was scored in accordance with previous protocols [5, 27] as negative (0), weak (1+), moderate (2+), or strong (3+) by two investigators. Scoring was performed blinded with respect to the histologic grade of PDAC specimens.

### Cell culture and cell treatments

The human pancreatic cancer cell lines CFPAC-1, BxPC-3, AsPC-1, SW1990 and PANC-1 were obtained from and validated by the Cell Bank of the Chinese Academy of Sciences (Shanghai, China) and maintained as per their instructions. The human breast cancer cell line MDA-MB-231 was kindly provided by Dr. Pei-Jun Liu (Medical College, Xi'an Jiaotong University), and maintained as described previously [43, 44]. The human glioma cell line U87MG was a kind gift from Dr. Mao-De Wang (Medical College, Xi'an Jiaotong University) and was cultured as previously described [45]. In some experiments, BxPC-3 and PANC-1 cells at the desired confluency (40–50%) were cultured for 24 h in serum-free media and then treated with recombinant mature human Nodal (rhNodal) (R&D Systems) and SB431542 (Sigma–Aldrich), an Alk4/5/7 inhibitor, or a DMSO vehicle control for another 24 h or 48 h. Recombinant Nodal and SB431542 were diluted according to the manufacturer's recommendations. The treatment concentration of SB431542 was 10 μM as previously reported [13, 21]. To knockdown Nodal expression, we used Nodal-targeted siRNAs. The siRNA for Nodal (oligo sequence: 5′-AGACAUGAUCGUGGAAGAAtt-3′) and the negative control siRNA (NC: 5′-CAUUUCGUCUGCCUCAUAUtt-3′) were purchased from GenePharm (Shanghai, China). Transfection was performed with LipofectamineRNAi MAX Reagent (Invitrogen, CA, USA) according to the manufacturer's instructions. The cells were used for further experiments 48 h after transfection.

### Isolation and culture of human pancreatic stellate cells

Normal pancreatic tissues (1.0–1.5 g) obtained from patients undergoing a pancreatic partial resection for benign pancreatic conditions at the First Affiliated Hospital of Xi'an Jiaotong University were immediately collected in sterile ice-cold Hanks balanced salt solution (HBSS) containing 100 U/ml penicillin and 100 μg/ml streptomycin (Gibco). The histological diagnostic assessment of specimens was confirmed by pathologists. Human pancreatic stellate cells (PSCs) were isolated using the density gradient method as previously described [46, 47]. Isolated PSCs were maintained at 37°C with 5% CO_2_ in DMEM/F12 (HyClone, Logan, USA) media supplemented with 10% heat-inactivated fetal bovine serum (FBS) (HyClone) and 100 U/ml penicillin and 100 μg/ml streptomycin. PSCs were identified by oil red staining of intracellular fat droplets and immunofluorescence of α-smooth muscle actin (α-SMA) and glial fibrillary acidic protein (GFAP) (see Supplementary Figure 1). Cells cultured in the above medium conditions for 24 h were used in additional experiments.

### Indirect co-culture of pancreatic cancer cells and PSCs

After pancreatic cancer cells were cultured in media supplemented with 10% FBS and grown to 50% confluence, the media was changed to contain 2% FBS and 100 U/ml penicillin and 100 μg/ml streptomycin. Two days later, cancer cell conditioned media was collected, centrifuged and filtered prior to incubation with isolated PSCs as previously described [48], and the PSCs were incubated with the conditioned media for up to 3 days.

### Quantitative real-time PCR

Total RNA was extracted from pancreatic cancer cells or PSCs using Trizol reagent (Invitrogen, CA, USA) according to the manufacturer's instructions. Reverse transcription was performed using a PrimeScript RT reagent Kit (TaKaRa, Dalian, China), and real-time PCR was performed with an iQ5 Multicolor Real-Time PCR Detection System (Bio-Rad, Hercules, CA, USA) using a SYBR Green PCR Kit (TaKaRa) according to the manufacturer's instructions. The primer sequences used are shown in Supplementary Table S2. For all real-time PCR analyses, β-actin was used to normalize RNA inputs.

### Western blotting analysis

Protein separation by SDS-PAGE and Western blotting were performed as previously described [24]. The primary antibodies used are listed in Supplementary Table S3. The density of specific protein bands was determined by QuantityOne image analysis software.

### Immunofluorescence

Immunofluorescence studies were performed as previously described [24]. Briefly, the cells were washed with phosphate-buffered saline (PBS), fixed with 4% paraformaldehyde for 10 min, permeabilized with 0.5% Triton X-100 for 5 min, and blocked with 1% bovine serum albumin for 1 h at room temperature. The cells were then incubated with a primary antibody at 4°C overnight. Following the primary antibody, incubation with a goat anti-rabbit dylight 594 (red) IgG antibody (QENSHARE BIOLOGICAL Inc., Xi'an, China) or a goat anti-mouse FITC (green) IgG antibody (ZSGB-BIO Inc., Beijing, China) at 1:200 dilutions for 1 h was performed at room temperature. Nuclei were stained with 4′,6-diamidino-2-phenylindole for 5 min. The cells were photographed with a fluorescent microscope (Nikon Eclipse Ti-s, Japan) using the appropriate excitation wavelength.

### Cell migration and invasion assays

To evaluate cell migration, wound-healing assays were performed according to previously reported protocols [49, 50]. Briefly, cells were seeded in fibronectin-coated 6-well plates, serum-starved overnight in media containing 1% FBS, and pre-treated as indicated for two days until reaching the appropriate confluence on the day of the experiment. The monolayers were then lightly scratched with a 200 μl or 1 ml pipette tip. Floating cells were washed off with PBS, and the remaining cells were cultured in serum-free media. Images of the same fields for each condition were visualized with a Nikon Eclipse Ti-S phase-contrast microscope with ×100 magnification at two preselected time points (0 h and 24 h). The wounded area was defined in each image by positioning green lines corresponding to the original scratch. The number of cells that migrated into the wounded area at 24 h was visually counted. The results (number of migrated cells) were presented as the mean ± SD of three independent experiments performed in triplicate. For cell invasion assessment, Transwell chamber assays were performed according to a protocol that was thoroughly described previously in one of our studies [24].

### Animal experiments

Twenty five-week-old female BALB/c nude mice were obtained from the animal center of the Medical College, Xi'an Jiaotong University, China. All animal experiments were performed according to the regulations established by the relevant Ethical Committee of the First Affiliated Hospital of Medical College, Xi'an Jiaotong University, Xi'an, China.

A reliable liver metastasis model of pancreatic cancer was established according to the methods described previously [51, 52]. The mice were anesthetized, a small left abdominal flank incision was created, and the extremitas inferior of the spleen was carefully exteriorized. BxPc-3 cells (5 × 10^5^) mixed with PSCs (1 × 10^5^) with viability greater than 90% were resuspended in 20 μl Ca^2−^ and Mg^2−^ free HBSSand inoculated subcapsularly into the spleen of each mouse with a 27-gauge needle. A vesicle appearing in the spleen was the criterion for successful inoculation. The spleen was replaced in the abdominal cavity, the peritoneum and skin were closed, and analgesia was administered. No surgery related fatalities occurred. The mice were then divided randomly into two groups: a control group receiving PBS treatment and the SB431542-treated group. Each group contained ten mice. After 1 week, SB431542 was used at 25 mg/kg by oral gavage twice daily for 4 weeks, similar to a previous report [21]. Mice were anesthetized and sacrificed 8 weeks after surgery. The liver, spleen and pancreas were harvested from each mouse. The primary tumor volume (mm^3^) was calculated as d^2^ × D/2, in which d and D represent the shortest and longest diameters, respectively. Livers were examined macroscopically and microscopically for the occurrence of metastases. The diameter of the largest liver metastasis was assessed. For each mouse liver, 6 sections were randomly acquired from evenly spaced areas through the liver, and the average number of metastases per mouse liver was calculated.

### Statistical analysis

All of the experiments were repeated at least three times. The results are expressed as the mean ± SD. Differences were evaluated using the Student's t-test or chi-Square test for multiple comparisons using SPSS (version 15.0; SPSS, Chicago, IL, USA). P-value < 0.05 was considered to be statistically significant.

## CONCLUSION

In this study, we found that Nodal is widely upregulated in pancreatic cancer cells and tumor-associated PSCs compared to normal pancreas. We presented clear evidence that Nodal induces signal transduction through the Smad2/3-dependent pathway. *In vitro*, we demonstrated that overexpression of Nodal promotes pancreatic cancer cell migration and invasion, induces EMT and enhances the expression of MMP2 and CXCR4. Furthermore, we provided evidence that blocking Nodal signaling activity with the small-molecule inhibitor SB431542 results in decreased number and size of liver metastases *in vivo*. Taken together, these results suggest that Nodal signaling is implicated in tumor progression and might be a therapeutic target for the treatment of pancreatic cancer.

## SUPPLEMENTARY FIGURES AND TABLES


