# Supplementary material for: Overexpression of Nodal induces a metastatic phenotype in pancreatic cancer cells via the Smad2/3 pathway
Source: Oncotarget. 2015 Feb 12;6(3):1490–506. doi: 10.18632/oncotarget.2686 (PMC4359309; doi:10.18632/oncotarget.2686)
Supplement: Supplementary file 1 [file oncotarget-06-1490-s001.pdf]

SUPPLEMENTARY FIGURES AND TABLES

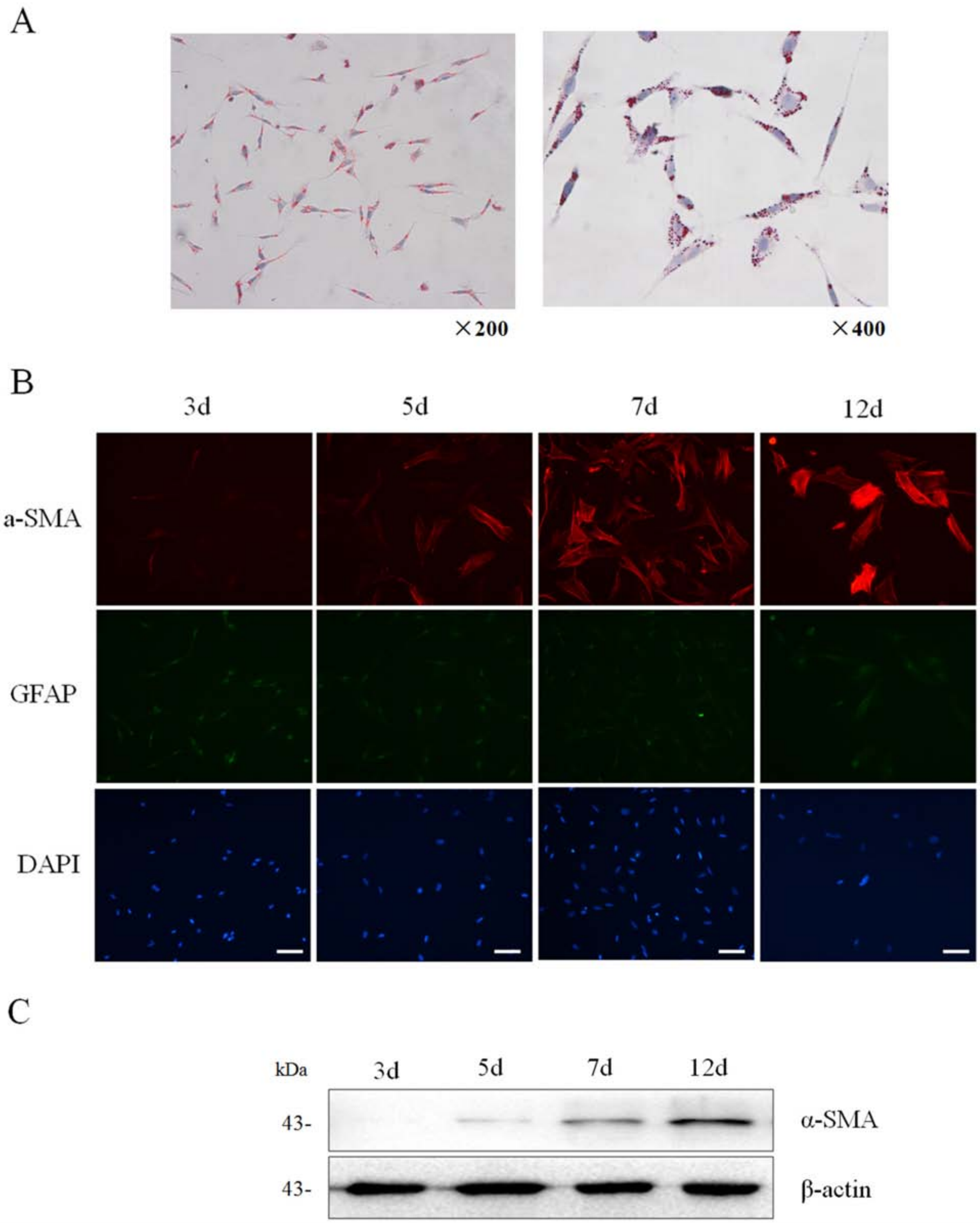

Supplementary Figure S1: Identification of isolated human pancreatic stellate cells (PSCs). PSCs isolated from normal pancreas tissue were maintained in DMEM/F12 media supplemented with 10% FBS and 100 U/ml penicillin and 100 μg/ml streptomycin as described in Materials and Methods. **(A)** Oil red staining of intracellular fat droplets in PSCs after 3 days in culture. **(B)** Immunostaining for α-SMA and GFAP in PSCs after 3 days, 5 days, 7 days and 12 days in culture. White scale bars, 50 μm. **(C)** Western blotting analysis of α-SMA expression in PSCs after 3 days, 5 days, 7 days and 12 days in culture. The results indicated that PSCs were gradually activated with the increasing time of culture.

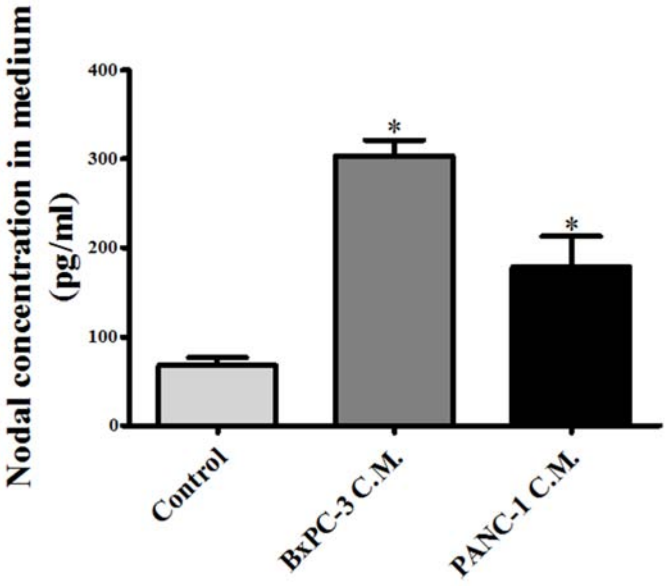

Supplementary Figure S2: The concentration of Nodal protein in the conditioned media was measured by ELISA Analysis. *$P < 0.05$ compared to the Control group.

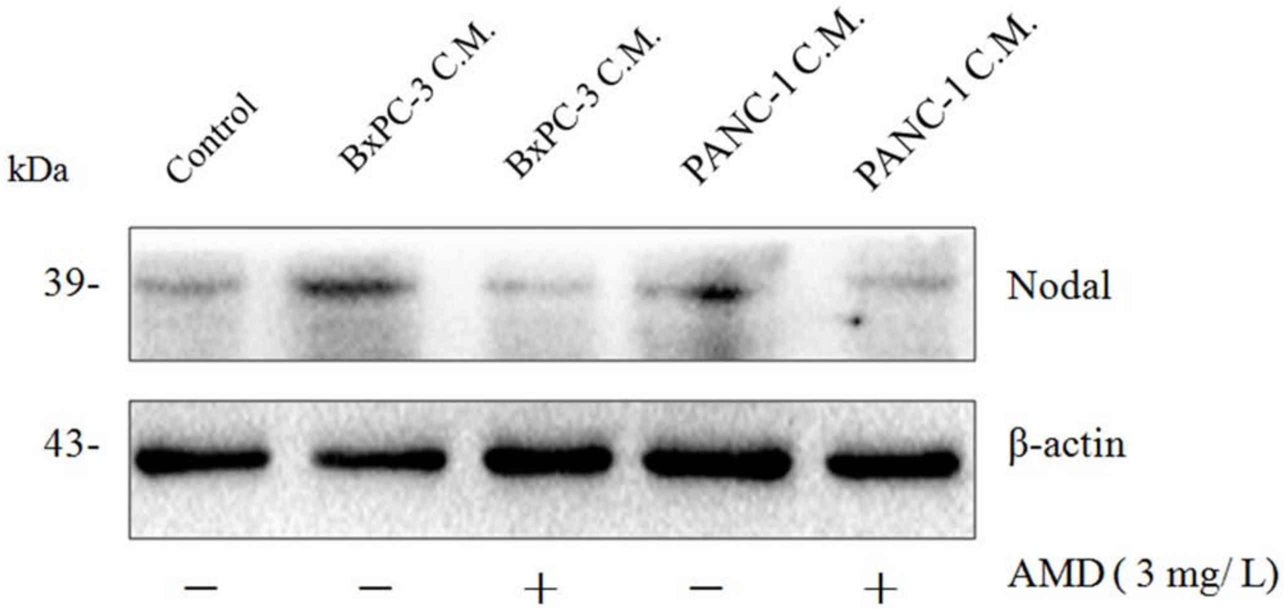

Supplementary Figure S3: The cancer cell C.M induced Nodal upregulation in PSCs were abrogated by Actinomycin D (AMD) (transcription inhibitor). PSCs were treated with cancer cell C.M. in the presence of AMD or not. After 48 h, the expression of Nodal in PSCs was detected by Western blotting analysis.

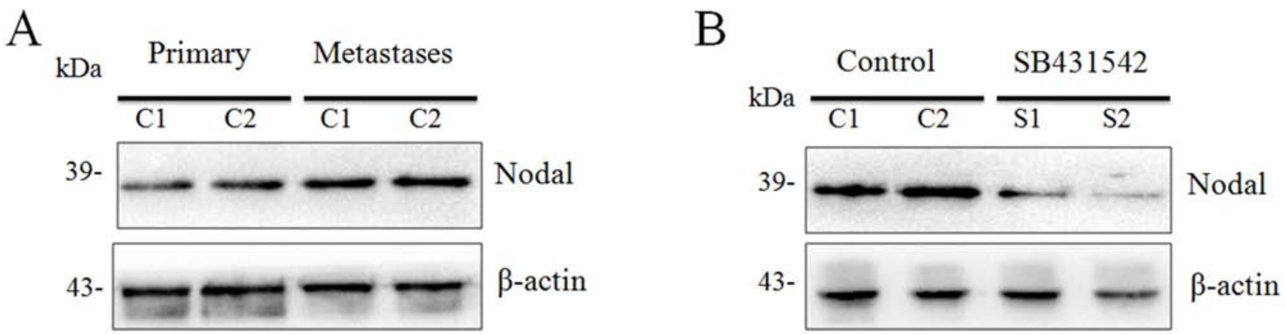

Supplementary Figure S4: The expression of Nodal in tumor tissues was detected by Western blotting analysis. (A) Western blotting analysis of Nodal expression in primary and metastases tumor specimens from Control group demonstrating that metastases tumor tissues exhibited higher Nodal expression compared to primary tissues, C1 and C2 represent the different mice samples in Control group respectively. **(B)** Western blotting analysis of Nodal expression in metastases tumor specimens from two groups demonstrating that SB431542 administration efficiently downregulated Nodal expression in metastases tumor tissues. C1 and C2 represent two metastases tumor specimens from two different mice in Control group, S1 and S2 represent two metastases tumor specimens from two different mice in SB431542 group.

Supplementary Table S1. The expression of Nodal in normal pancreas, chronic pancreatitis and pancreatic cancer

Classification	Nodal expression in pancreatic cancer cells (N)				Chi-Square (*P*-value)
	0+	*1+*	*2+*	*3+*	
**Normal pancreas**	21	2	0	0	< 0.0001
**Chronic pancreatitis**	15	6	3	0	
**Pancreatic cancer**	26	33	23	13	
Poor	5	3	6	7	
Moderate	9	11	10	4	0.002
Well	12	19	7	7	

Supplementary Table S2. The expression of Nodal in pancreatic cancer

Nodal expression in tumor-associated stroma	Nodal expression in pancreatic cancer cells (N)			Chi-Square (*P*-value)
	0+ or *1*+	*2*+ or *3*+	Total	
**Negative**	51	17	68	< 0.001
**Positive**	8	19	27	
**Total**	59	36	95	

Supplementary Table S3. Primers for real-time PCR

Genes	Forward primer (5′-3′)	Reverse primer (5′-3′)
Nodal	TGCTCCTTATGCTCTACTCCAA	TGACCTTCCGACACAGTTGA
Cripto	GAGTGCTGAAGGAATGGAAGTC	CACCACCTAATCACCAATCAAGTA
Alk7	AAGCAACACCTCAACTCATCTT	CACCTCACTTACATCTTCCACTAA
Alk4	CCTGTTCCTCATCATCATCATTG	CGTCTGGTATATCTCTGCTTCC
E-cadherin	ATTCTGATTCTGCTGCTCTTG	AGTCCTGGTCCTCTTCTCC
N-cadherin	ACAACAGACCTGAGTTCTTACAC	TTGGAGCCTGAGACACGATT
Vimentin	AATGACCGCTTCGCCAAC	CCGCATCTCCTCCTCGTAG
Snail	CTTCTCCTCTACTTCAGTCTCTTC	CGTGTGGCTTCGGATGTG
MMP2	GATGATGCCTTTGCTCGTGC	CAAAGGGGTATCCATCGCCA
CXCR4	TGACGGACAAGTACAGGCTGC	CCAGAAGGGAAGCGTGATGA
β-actin	CATCACTATCGGCAATGAGC	GACAGCACTGTGTTGGCATA

Supplementary Table S4. A list for the utilized primary antibodies

Antibody	Dilution & Use	Company
Mouse anti-Nodal	1:1000(WB)<br>1:150(IHC/IF)	Abnova
Rabbit anti-Cripto	1:1000(WB)	Abcam
Rabbit anti-Alk7	1:1000(WB)	Epitomics
Rabbit anti-Alk4	1:1000(WB)	Epitomics
Rabbit anti-pSmad2	1:1000(WB)	Cell Signaling Technology
Rabbit anti-Smad2	1:1000(WB)	Cell Signaling Technology
Rabbit anti-E-cadherin	1:1000(WB)	Santa Cruz Biotechnology
Rabbit anti-N-cadherin	1:500(WB)	Santa Cruz Biotechnology
Rabbit anti-Vimentin	1:1000(WB)	Bioworld
Rabbit anti-Snail	1:500(WB)	Santa Cruz Biotechnology
Rabbit anti-MMP2	1:800(WB)	Bioworld
Rabbit anti-CXCR4	1:1000(WB)	eBioscience
Rabbit anti-α-SMA	1:200(IF)	Bioworld
Mouse anti-GFAP	1:50(IF)	Santa Cruz Biotechnology
Mouse anti-β-actin	1:5000(WB)	Santa Cruz Biotechnology
